# *VdCYC8*, Encoding CYC8 Glucose Repression Mediator Protein, Is Required for Microsclerotia Formation and Full Virulence in *Verticillium dahliae*

**DOI:** 10.1371/journal.pone.0144020

**Published:** 2015-12-03

**Authors:** Zhi-Fang Li, Yi-Jie Liu, Zi-Li Feng, Hong-Jie Feng, Steven J. Klosterman, Fang-Fang Zhou, Li-Hong Zhao, Yong-Qiang Shi, He-Qin Zhu

**Affiliations:** 1 State Key Laboratory of Cotton Biology, Institute of Cotton Research of Chinese Academy of Agricultural Sciences, Anyang, Henan, 455000, China; 2 USDA-ARS, Salinas, California, 93905, United States of America; Nanjing Agricultural University, CHINA

## Abstract

*Verticillium dahliae* is the primary causal agent for Verticillium wilt disease on a diverse array of economically important crops, including cotton. In previous research, we obtained the low-pathogenicity mutant T286 from the T-DNA insertional mutant library of the highly virulent isolate Vd080 derived from cotton. In this study, the target disrupted gene *VdCYC8* was identified by TAIL-PCR, encoding a homolog of CYC8 proteins involved in glucose repression. The deletion mutant *ΔCYC8* exhibited several developmental deficiencies, including reduced microsclerotia formation, reduced sporulation, and slower growth. Moreover, compared with the wild type strain Vd080, the pathogenicity of strain Δ*CYC8* was significantly decreased on cotton seedlings. However, the complementary mutants *ΔCYC8*-C led to restoration of the wild type phenotype or near wild type levels of virulence on cotton. Interestingly, pathogenicity of the strains was correlated with *VdCYC8* gene expression levels in complemented mutants. Gene expression analyses in the wild type strain Vd080, the Δ*CYC8*-45 strain, and complemented strain Δ*CYC8*-C26 indicated that *VdCYC8* regulates the transcription levels of several genes in *V*. *dahliae* that have roles in melanin and production.

## Introduction

*Verticillium dahliae* Kleb. is a soil-borne fungus that causes Verticillium wilt disease on plants worldwide. The melanized microsclerotia produced by *V*. *dahliae* can survive over 10 years in the absence of a host, and therefore pose a long-term threat to more than 200 plant species, including several economically important crops such as cotton, potato, strawberry, lettuce [1–4]. Many isolates of *V*. *dahliae* lack host specificity and can be disseminated among different regions and hosts [5]. The microsclerotia germinate under favorable conditions, penetrate plant cells, and systemically spread and colonize in the vascular tissues [6]. Once infection with *V*. *dahliae* occurs, chemical control agents are useless, owing to the inaccessibility of the fungal propagules within the plant [2, 7]. Though some crop rotations reduce Verticillium wilt incidence these are largely ineffective due to the long term survival of the microsclerotia of V. dahliae, and can be difficult to apply for multiple susceptible hosts in specific areas [8–10]. For these reasons, microsclerotia of *V*. *dahliae* have been considered as the primary targets for controlling this systematically vascular disease [11]. However, key developmental events in the disease cycle of *V*. *dahliae* remain unresolved.

Virulence in fungal pathogens is controlled by a network of signaling pathways, which must occur in concert with other complex signaling events [12]. Multiple genes of *V*. *dahliae* have been identified as involved in microsclerotial development, sporulation, growth, stress and starvation tolerance, cell-wall degradation, and pathogenicity. The sucrose nonfermenting protein kinase gene *VdSNF1* was required for the virulence on tomato and eggplant, which regulated catabolic repression and the hydrolytic cell wall-degrading enzyme activities in *V*. *dahliae* [13]. Disruption of the kinase encoding gene *VMK1* in two *V*. *dahliae* isolates resulted in severely reduced virulence on twelve different hosts. Furthermore, conidiation and microsclerotia formation were inhibited [14]. The hydrophobin gene *VDH1* is critically important for microsclerotia development and spore desiccation tolerance, but not for virulence on tomato [15, 16]. Overexpression of *VdTHI4* promoted stress tolerance in *V*. *dahliae*, including to UV-damage and oxidative stress; whereas deletion mutants displayed severely reduced biomass in host tissues and caused no disease symptoms [17]. With the advent of digital gene-expression profiling and RT-qPCR analysis, available evidence suggests a series of C_2_H_2_ zinc finger-encoding genes participate in fungal growth, microsclerotia formation, various stress responses, and virulence in *V*. *dahliae* [18]. In addition, *VGB*, *VdUDG*, *Sge1*, and *VdPR3* are involved in multiple signaling pathways in regulating pathogenicity, sporulation, and formation of microsclerotia in *V*. *dahliae* [19–22].

Much of the available information on the function of CYC8 was developed in the model fungus, *Saccharomyces cerevisiae*. In *S*. *cerevisiae*, CYC8 is a general transcriptional repressor that acts in a co-repressor complex with Tup1, and regulates many developmental processes, including nutrient utilization, osmotic stress, meiosis, mating, and sporulation [23–26]. The CYC8 or CYC8-TUP1 co-repressor complex exerted various effects on glucose repression for maltose metabolism, a global regulatory system in *S*. *cerevisiae* [27]. In other fungi, SSN6 is a crucial regulator of morphological transition and virulence independent of TUP1 in *Candida albicans* [28], although SSN6 may interact with histone deacetylase Rpd31 and play dual roles in filament development in *C*. *albicans* [29]. *MoTup1* was identified as the requirement for growth, conidiogenesis and pathogenicity in the rice blast fungus, *Magnaporthe oryzae* [30]. In the model *Neurospora crassa*, Rco1, the homologue of Tup1, functions in growth and development [31]. However, the role of CYC8 has not been elucidated in *V*. *dahliae*.

In our previous work, 25 weakly pathogenic isolates were obtained by insertional mutagenesis of strain Vd080 from cotton after two-rounds of pathogenicity screening [32]. The mutant strain T286, displaying a single-copy T-DNA insertion, was significantly reduced in virulence and also exhibited other defects, including slower growth *in vitro*, reduced sporulation, and no melanin or microsclerotia formation.

The objectives of this study were to determine the specific gene disruption responsible for the mutant phenotype of strain T286 of *V*. *dahliae* and to further assess the mutant phenotype, including potential roles in fungal development, pathogenicity on cotton plants, and the influence on the transcriptional regulation of several genes implicated in melanin production and microsclerotia formation.

## Materials and Methods

### Fungal strains and growth conditions

The virulent defoliating and microsclerotia-forming *V*. *dahliae* wild type strain Vd080, derived from typical Verticillium-wilt-symptomatic cotton in Hebei province of China, was used in this study. This isolate and all genetic mutants generated in this study were single-spore purified, and stored in 20% glycerol at -80°C. The fungus was cultured on potato dextrose agar (PDA), and the appropriate antibiotics were added for selection of mutants. The concentration of both hygromycin B and chlorimuron in the media was 50 ug/ml. Conidia production for pathogenicity assays and phenotypic analysis was performed in liquid Czapek-Dox medium as previously described [20].

### Gene isolation, cloning and phylogenetic analysis

The virulence-deficient mutant T286 was identified as a single-copy T-DNA integrant [32]. To isolate the target disrupted gene in this current study, a thermal asymmetric interlaced PCR (TAIL-PCR) was conducted with three specific primers on each border [32], and four arbitrary degenerate primers [33]. The specific tertiary PCR products of the appropriate primer combinations were cloned for sequencing. The specific T-DNA insertion site in T286 was identified by comparing the cloned sequences against the available genome sequences of VdLs.17 using BLASTn searches [34].

Thirty-five CYC8 homologous protein sequences from twenty-nine different fungi were identified by BLASTp analysis and downloaded for phylogenetic analysis. Phylogenetic analysis was performed using Mega v.5.1 after multiple alignment of the data by CLUSTAL_X [35], with gaps treated as missing data. Clustering was performed using the neighbor-joining method [36]. Bootstrap analysis was used to evaluate the tree topology of the neighbor-joining data by performing 1000 re-samplings [37].

### Vector construction and fungal transformation

A two-step method was used to construct the CYC8 knock out vector, including fusion fragment generation and a gateway cloning reaction. Specific primer pairs CYC8-P1/P3 and CYC8-P2/P4 (Table 1) were designed to amplify the *CYC8* upstream (UP) and downstream (DOWN) Vd080 genomic DNA from *V*. *dahliae*. Primers Hyg-F/R (Table 1) were designed for amplification of the hygromycin phosphotransferase cassette (*HPH*, conferring resistance to the antibiotic hygromycin B), obtained from plasmid pCTHyg []. CYC8-P2 and P3 (Table 1) possess adaptors for the reverse complemented sequence of Hyg-F and Hyg-R. Fusion PCR was performed using the DNA fragments of UP, HPH and DOWN together in one reaction. The CYC8 fusion fragment was amplified via nested PCR with primers CYC8-P2/P5 supplementing the adaptor of attB at the 5′end (S1A Fig). The binary vector pGKO_2_-Gateway carrying the lethal gene *HSVtk* was applied for the gateway reaction [38]. With the aid of recombination between attB and attP, the CYC8 fusion fragment was cloned into the binary vector to generate the CYC8 knock out vector, pGKO_2_-CYC8 (S1B Fig).

**Table 1:** Primers used in vectors construction in this study.

Primer	Primer sequence (5’-3’)	Expected length
Hyg-F	TTGAAGGAGCATTTTTGGGC	1.8 kb
Hyg-R	TTATCTTTGCGAACCCAGGG	
CYC8-P1	TCCATTCCTGAGACCCGCAC	1.1 kb
CYC8-P3	^a^ GCCCAAAAATGCTCCTTCAAGAGGGACTGAGGGCATCGAG	
CYC8-P4	^b^ CCCTGGGTTCGCAAAGATAA CGTTGTCCGCCAACCATCTG	1.2 kb
CYC8-P6	TGTAGAAGCGACGACGGACC	
CYC8-P2	^c^ GGGGACAAGTTTGTACAAAAAAGCAGGCTTCCTGTCGCCAACTCGATCC	4.1 kb
CYC8-P5	^d^ GGGGACCACTTTGTACAAGAAAGCTGGGTATGCCAGCCTTGTAGCCCAT	
CYC8COM-F	^e^ GAATTCTCCTGTCGCCAACTCGATCC	4.3 kb
CYC8COM-R	^f^ CTTAAGATGCCAGCCTTGTAGCCCAT	

^a,b^ The underlined adaptors were the reversed complemented sequence of Hyg-F and Hyg-R;

^c,d^ the wavy line regions indicate attB1 and attB2 adaptors for Gateway BP reaction;

^e,f^ The underlined adaptors indicated the recognition sequence of endonuclease *Eco*R1 and *Afl*II.

The binary vector pSULPH-gfp encoding green florescent protein (GFP) and the chlorimuron-resistance marker was used as the backbone for CYC8 complementary vector construction. The CYC8 functional fragment composed of promoter, coding region, and terminator was amplified by the primer pair CYC8COM-F /CYC8COM-R with *Eco*R1 and *Afl*II recognition sites at the 5’ end, respectively (Table 1). The complementary vector COM-CYC8 was generated by ligation between the backbone vector and functional CYC8 fragment.

*Agrobacterium tumefaciens* isolate AGL1 carrying the CYC8 knock out binary vector pGKO_2_-CYC8 was transformed into wild-type isolate Vd080, while the complementary vector COM-CYC8, containing the wild type *CYC8*, was introduced into strain T286 by an *Agrobacterium tumefaciens*-mediated transformation (ATMT) method described previously [20].

### Positive mutants screening and verification

To confirm deletion mutants, two pairs of primers were applied for mutant screening. Hyg-F/R was used to identify successful replacement of target gene *VdCYC8* with the hygromycin-resistance cassette. The other pair of tested primers originated from the *VdCYC8* coding region; CYC8test-F (5’-GCGTTCGAAAAGGCCAACGA-3’) and CYC8test-R (5’-CGTTGGTCATCGAATCGGCG-3’) yield a PCR amplicon of 2.1 kb if wild type *CYC8* is present. The positive *VdCYC8* deletion mutants possessed an HPH-specific band when extracted DNA was amplified using the primer pair Hyg-F/R, but not the wild type *VdCYC8* coding region, which could be amplified with primer pair CYC8test-F/R.

To investigate the transcriptional profile of *VdCYC8* in both Vd080 and deletion mutant strains, total RNA was isolated from 7-day-old mycelia of each strain using the RNAsimple kit in accordance with the manufacturer’s instructions (Tiandz Inc., Beijing, China). Total RNA was treated with DNase1 (Invitrogen, CA, USA) to remove DNA contamination. The DNA concentration was measured with a Nanodrop 2000 (Thermo Scientific Corp., MA, USA). Five hundred nanograms of total RNA was used in reverse-transcription for first-strand cDNA synthesis with oligo (dT) primer according to SuperScript^®^ III First Strand Synthesis kit directions (Invitrogen, USA). Oligonucleotide primers flanking exon-intron junctions were specifically prepared for both *VdCYC8* and housekeeping gene β-tublin (*Bt*) as follows: CYC8RT-F (5’-GGATGCCCTCGATGCTTACT-3’) CYC8RT-R (5’-CGTCGCTGATCTGGTTGTTG-3’) and VerBt-F(5’-GACTTCCGTAACGGTCGCT-3’) and VerBt-R (5’-TTCTTGCTCTGGACGTTGCG-3’).

For mutant complementation, primers CYC8COM-F/R were used to verify the presence of wild type *CYC8*, and the chlorimuron-resistance cassette with primers Sul-F (5’-TCGACGTGAGAG CATGCAATTC-3’) and Sul-R (5’-GTCGAGGTGCCAACGCC ACAGT-3’). Both genes should be detectable in the genomic DNA of positive complementary mutants. Levels of *CYC8* transcriptional expression from Vd080 and complemented mutant strains were assessed by reverse transcription quantitative PCR (RT-qPCR) analysis as described below. The complementation vector carried a GFP marker, the mycelia of the T286 and GFP-transformed Vd080 strains were observed under fluorescence microscopy.

Southern blot analysis was conducted to further identify the positive mutants by the presence or absence of the *VdCYC8*. The genomic DNA of the mutants and Vd080 was digested by EcoR I and separated on a 0.7% agarose gel, and transferred to a nylon membrane (Roche). The probe was amplified from *VdCYC8* with the primer CYC8SB-F 5’-GCAGCCTCG GTACGCAAATC-3’ and CYC8SB-R 5’-AGGTACCAGCTCTGTGCG TC-3’. The probe was labeled with digoxigenin (DIG) (Roche, Germany) according to the manufacturer’s instructions. Hybridization and detection was performed according to the manufacturer instructions (Roche).

### Phenotypic analysis

Developmental and morphological characteristics of both *CYC8* deletion (Δ*CYC8*) and complementary mutants (Δ*CYC8-C*) were investigated and compared between *V*. *dahliae* strain Vd080, transformants of Vd080, and strain T286. The phenotypic analyses included microsclerotia formation, conidia-production, radial growth on PDA, and spore germination rate. The photos of the mutants and wild type were taken post 9 days inoculation using an Olympus microscope, CX 21 (Japan). Phenotypic analyses were performed following methods of Zhang et al. [20] and Tzima et al. [19].

### Pathogenicity assays

Verticillium wilt susceptible cotton variety Jimian 11 was used for pathogenicity assays of the genetically transformed mutant strains derived from Vd080. Fungal inoculation, disease investigation, and data analyses were conducted as described previously [39]. A disease index (DI), as previously described [39], was used to evaluate the disease severity of cotton seedlings and pathogenicity of each strain.

### Reverse-transcription quantitative Real-Time PCR

To monitor the transcriptional expression profile of melanin and microsclerotia production -related genes in the wild type Vd080 and mutant strains, the gene loci of *VDAG_00189*, *VDAG_00190*, *VDAG_00184*, *VDAG_03665*, *VDAG_03393*, *VDAG_04954* were selected for quantitative real-time PCR (RT-qPCR) analysis (Table 2) [40]. The expression patterns of *VdCYC8* at different stages of development were also assessed by RT-qPCR with *V*. *dahliaeβ-tubulin* as the reference gene. Mycelia of Vd080 were harvested at 4, 8, 12, 16, and 20 days for total RNA isolation.

**Table 2:** qRT-PCR primer information of six microsclerotia-formation related genes in *V*. *dahlia*.

Gene ID	Predicted function	Primer sequence (5’-3’)	Annealing temperature	Amplification length
VDAG_00189	laccase	F:GCTACCGCCAGGATCAACCA	62°C	158 bp
		R:CCTCATCGTACTGCCCGACA		
VDAG_00190	conidial yellow pigment biosynthesis polyketide synthase	F:ATGTCAACAAGGCGCTCCAAAG	60°C	142 bp
		R: AAATTGCTTTCCACCAACGCCT		
VDAG_00184	amino acid adenylation/polyketide synthase	F: TCCTCGATCTCATTCAGCTGGC	62°C	191 bp
		R: GGACTTGAAATAGCCGTGCTGG		
VDAG_03665	tetrahydroxynaphthalene reductase	F:ACATTGTCTGCTCAAACAGCGG	59°C	144 bp
		R:CACCCTCTTCGAGGTGCTTGTA		
VDAG_03393	scytalone dehydratase	F: ATCACCTTCGACGACTACCTCG	63°C	153 bp
		R: CATGGCCTCCCAGATCTTGTCT		
VDAG_04954	pigment biosynthesis protein Ayg1	F: GATGGGCACGAGTATCCGTTTC	60°C	80 bp
		R: GTCTTGTACTCCGCCACAGTCT		

RNA isolation and cDNA synthesis were performed as mentioned above. RT-qPCR was performed in a LightCycler 480 (Roche, Germany) using SYBR Green I (Bio-Rad, USA). Total volume of PCR reaction was 20 ul, including 10 ul of 2×SYBR Green I SuperMix, 0.2 ul of each primer (Table 2), and 2-ul cDNA of *V*. *dahliae*. The reaction profile was performed as an initial 95°C for 5 min, followed by 40 cycles of 95°C for 15 s, appropriate annealing temperature as shown in Table 2 for 15 s and 72°C for 30 s. RNA from each strain was collected from three biological replicates, and three technical replicate reactions were subsequently run for each biological replicate.

RT-qPCR efficiency was determined by 10-fold gradient dilutions of cDNA of each target sequence for standard curve production. Under the optimal annealing temperature, the calculated efficiency of all primers was 90%–105%. β-tublin of *V*. *dahliae* was used as reference gene for calibration in all the experiments. Melt curve analyses were carried out to evaluate the primer dimers. Relative expression of each gene was determined from cycle threshold (C_T_) values according to the 2^-ΔΔCT^ method [41].

## Results

### Cloning, expression pattern and phylogenetic analysis of *VdCYC8*

**Fig 1:**
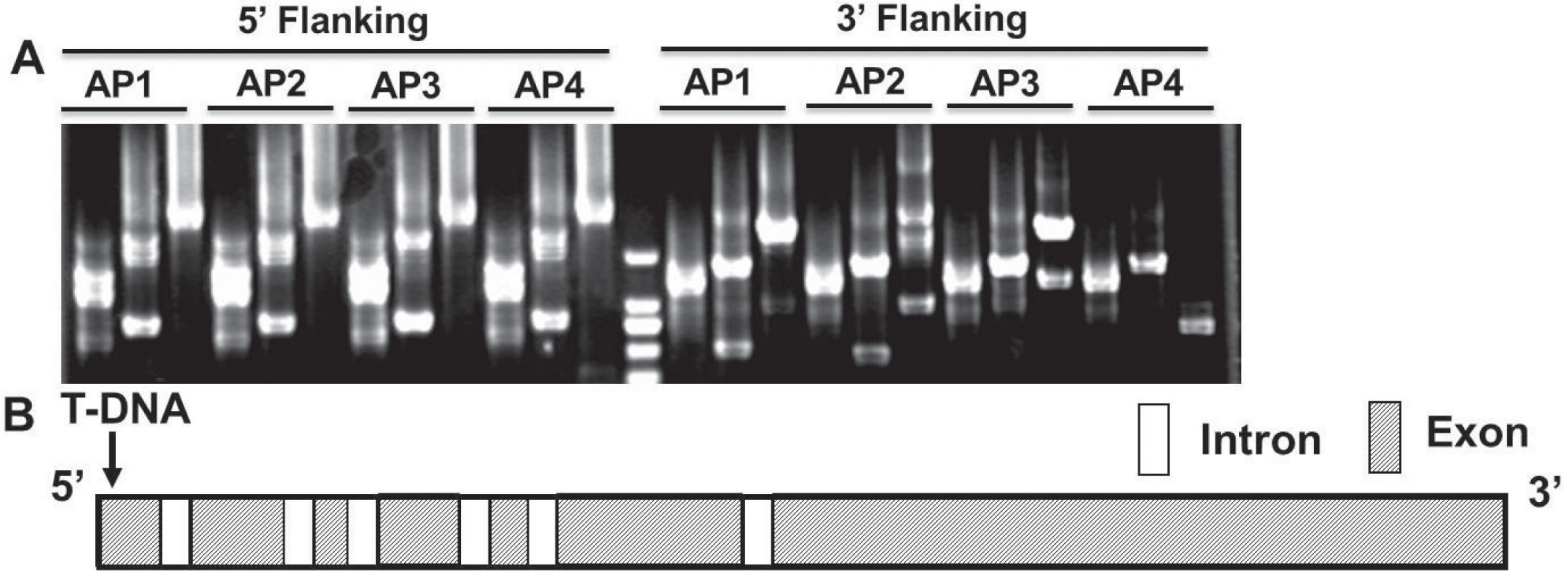
TAIL-PCR and identification of the T-DNA insertion site in mutant T286 of *Verticillium dahliae*. **A)** Electrophoresis of TAIL-PCR amplification products obtained from the application of four arbitrary degenerate primers, and three specific primers at each T-DNA border. PCR products from the different combinations (AP1-4) were cloned and sequenced. **B)** Gene structure of *VdCYC8* and the insertional position of T-DNA in T286.

In the ATMT insertional mutant strain T286, both T-DNA flanks were successfully identified as *VdCYC8* using the appropriate combination of gene-specific primers and four arbitrary degenerate primers (Fig 1A). The disrupted gene showed high similarity with gene locus *VDAG_07052* originating from strain VdLs.17 [34]. The T-DNA was integrated with the first exon of gene *VDAG_07052* in T286, as evident by comparison of the sequence to that of the reference genome of strain VdLs.17. *VDAG_07052* consists of seven exons and six introns with a full length of 3201 bp, encoding a CYC8 glucose repression mediator protein (Fig 1B).

**Fig 2:**
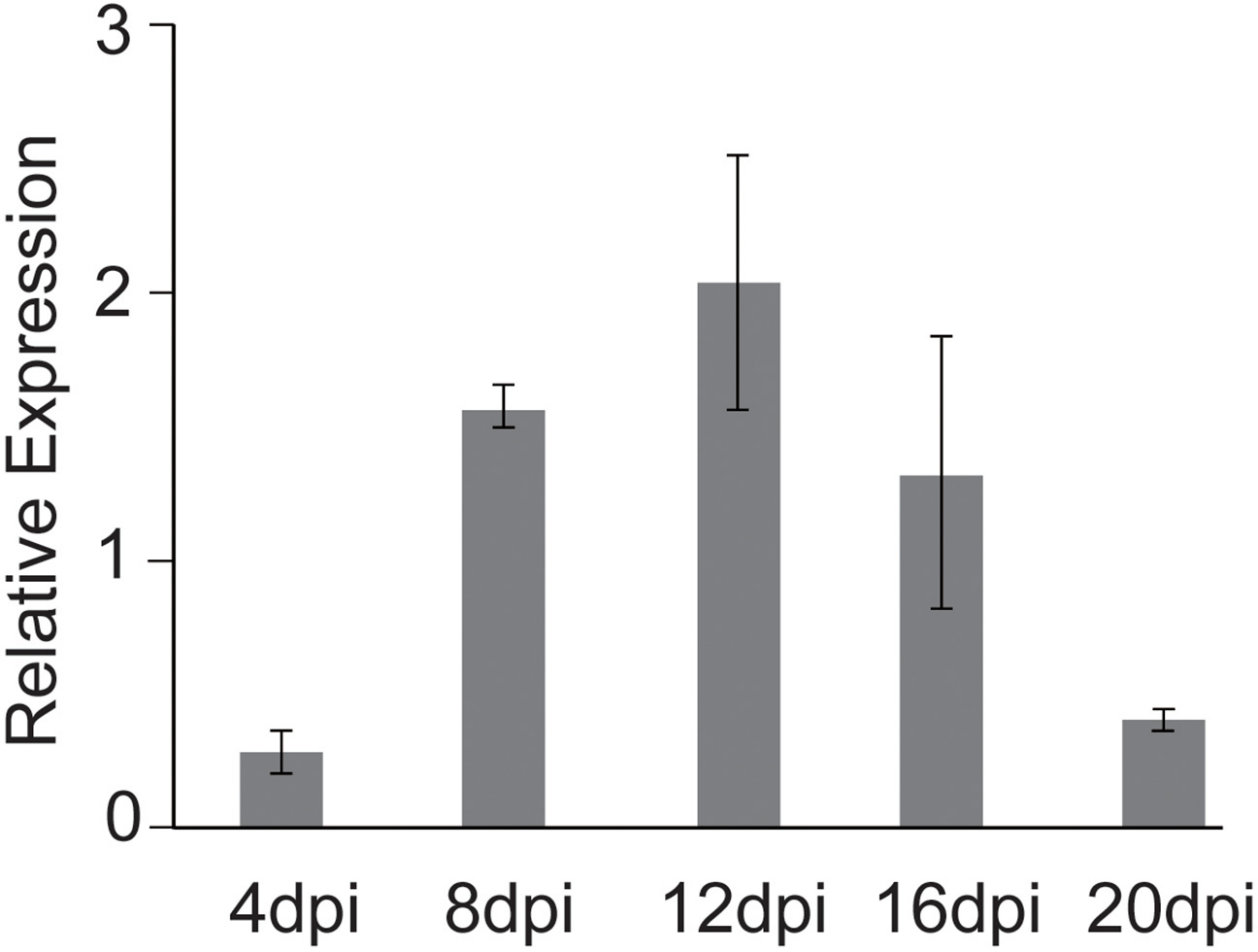
Transcriptional expression levels of *VdCYC8* in strain Vd080 of *Verticillium dahliae*, at different developmental stages. The *V*. *dahliae B-tubulin*, amplified using primers VerBt-F/R, was employed as the reference gene in the analyses. The mycelial phase of Vd080 was harvested at 4, 8, 12, 16, and 20 days post inoculation (dpi) for analyses of relative expression.

The analysis of *VdCYC8* relative expression levels at different cultivated periods revealed increased in the early stage and reached maximum at twelve days of growth. However, *VdCYC8* expression was subsequently reduced, at remaining time points examined (Fig 2).

Furthermore, phylogenetic analysis indicated that VdCYC8 showed the closest relationship with that of the homolog from a closely related species, *Verticillium longisporum and V*. *alfalfae*, followed by *Colletotrichum sp*. (S2 Fig). VdCYC8 also shared similarity in amino acid sequence of CYC8 from another major wilt fungus, *Fusarium oxysporum* (S2 Fig).

### Selection and verification of *VdCYC8* replacement and mutant complementation

*VdCYC8*-specific upstream (1.1 kb) and downstream (1.2 kb) DNA fragments were amplified from DNA of *V*. *dahliae* strain Vd080 with primers CYC8-P1/P3 and CYC8-P2/P4, respectively. The selectable marker gene, encoding hygromycin phosphotransferase (1.8 kb), was amplified from plasmid pCTHyg with primers HygRH-F/R. The *VdCYC8* fusion fragment was produced from the fusion of the upstream *VdCYC8* flanking sequence, the Hyg resistance marker, and the downstream VdCYC8 flanking sequence fragments. Then, nested PCR was conducted by primers CYC8-P2/P5 with the adaptor of attB at the 5’ for the gateway reaction (S1 and S3 Figs). The *VdCYC8* knockout vector pGKO_2_-CYC8 carrying the lethal gene *HSVtk* was generated by gateway reaction (S1B Fig).

**Fig 3:**
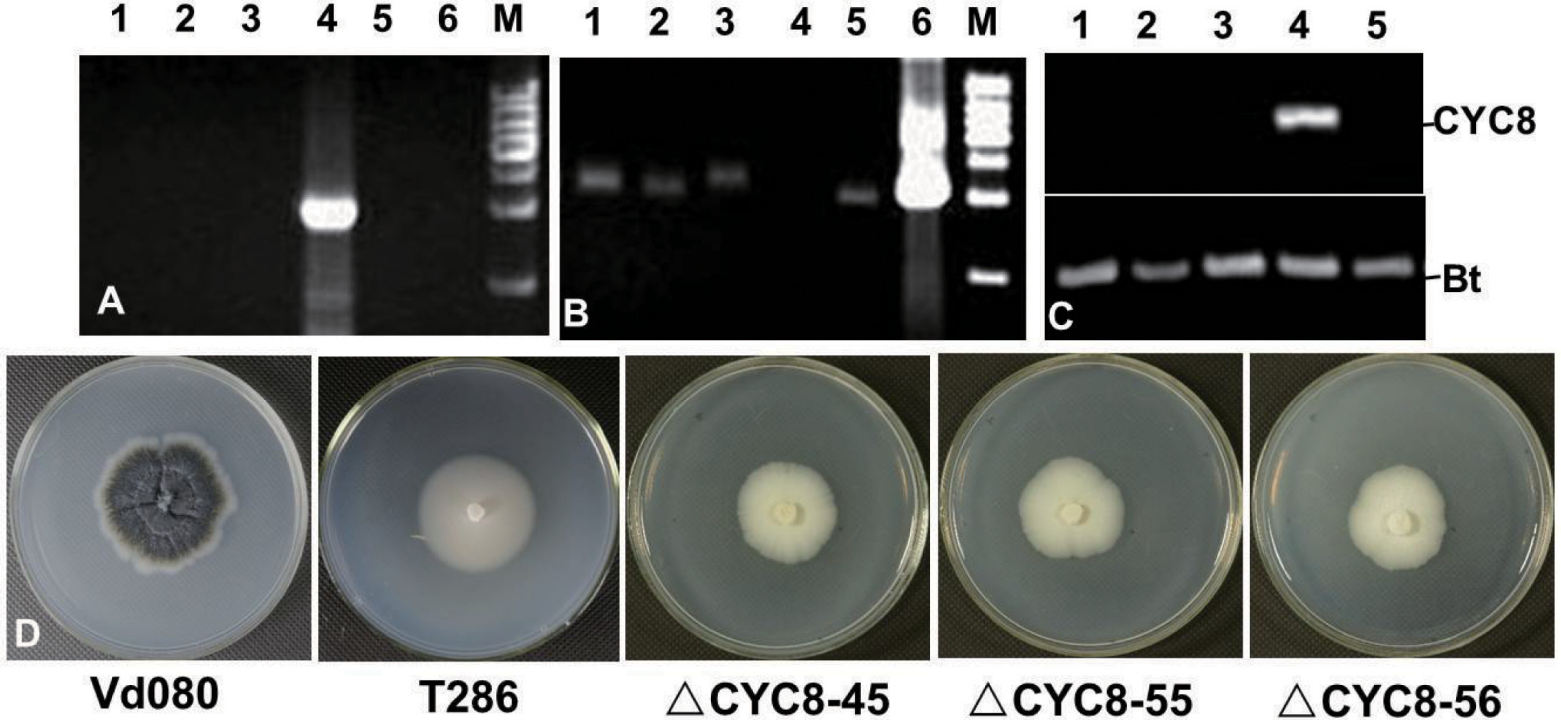
Molecular verification of *VdCYC8* deletion mutants, and comparison of *in vitro* growth of wild type strain Vd080 and the *CYC8* deletion mutant strains (Δ*CYC8-*45, Δ*CYC8-*55, Δ*CYC8-*56). **A)** PCR verification of *vdCYC8* deletion mutants with primer CYC8test-F/R. Lanes 1–6 indicate PCR from DNA template extracted from strains Δ*CYC8*-45, Δ*CYC8*-55, Δ*CYC*8-56, Vd080, T286, and the positive control pGKO_2_-CYC8 plasmid, respectively. The molecular weight marker (M) is 1kb ladder. **B)** Tested with primer Hyg-F/R. **C)** Reverse-transcription PCR analysis of *CYC8* expression in strains Δ*CYC8*-45 (lane 1), Δ*CYC8*-55 (lane 2), Δ*CYC8*-56 (lane 3), Vd080 (lane 4), T286 (lane 5), respectively. The *V*. *dahliae* β-tubulin (Bt), amplified using primers VerBt-F/R, is shown as a control. **D)** Characteristics of growth of different isolates of the *V*. *dahliae CYC8* mutant strains on PDA.

Seventeen *VdCYC8* deletion mutant strains were selected, single-spored, and cultivated for three generations. Among these, the deletion mutants Δ*CYC8-*45, Δ*CYC8-*55 and Δ*CYC8-*56 were used for verification of *VdCYC8* deletion and further investigation (Fig 3D). No ectopic mutants were identified with the lethal gene *HSVtk*. The Hyg selectable marker cassette was positively identified from DNA extracted from all three knockout mutant strains Δ*CYC8*-45, Δ*CYC8*-55 and Δ*CYC8-*56, but not from Vd080 (Fig 3A and 3B). In contrast to wild type strain Vd080, reverse transcription quantitative PCR analysis revealed lack of gene CYC8 expression in strains Δ*CYC8*-45, Δ*CYC8*-55 and Δ*CYC8*-56 (Fig 3C). This confirmed *VdCYC8* replacement with the Hyg selectable marker cassette in the three knockout mutants examined.

**Fig 4:**
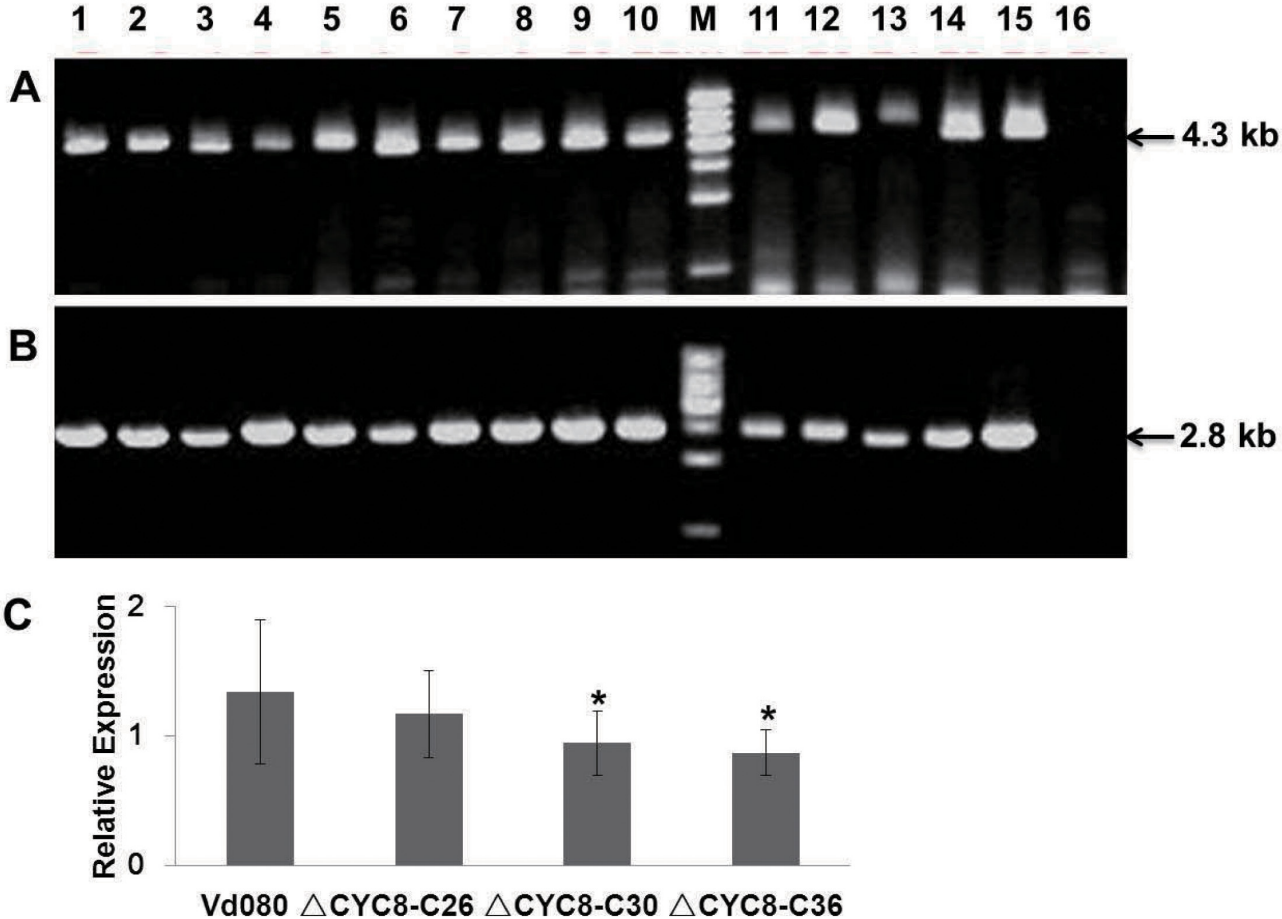
Identification of *VdCYC8*-complemented mutants in *Verticillium dahliae* by PCR identification and *VdCYC8* relative expression analysis. **A)** Confirmation of the presence of the wild-type complemented *VdCYC8* fragment. **B)** Confirmation of the presence of the chlorimuron cassette in the Δ*CYC8*-C strains. Lanes 1–15 indicate PCR results using primer Sul-F/R and DNA template obtained from the different complemented mutant strains, respectively; Lane 16 represents T286. **C)** Relative expression analysis of *VdCYC8* using primer CYC8RT-F/R. *Indicates a significant difference between mutant and the wild-type strain Vd080 (P<0.05).

**Fig 5:**
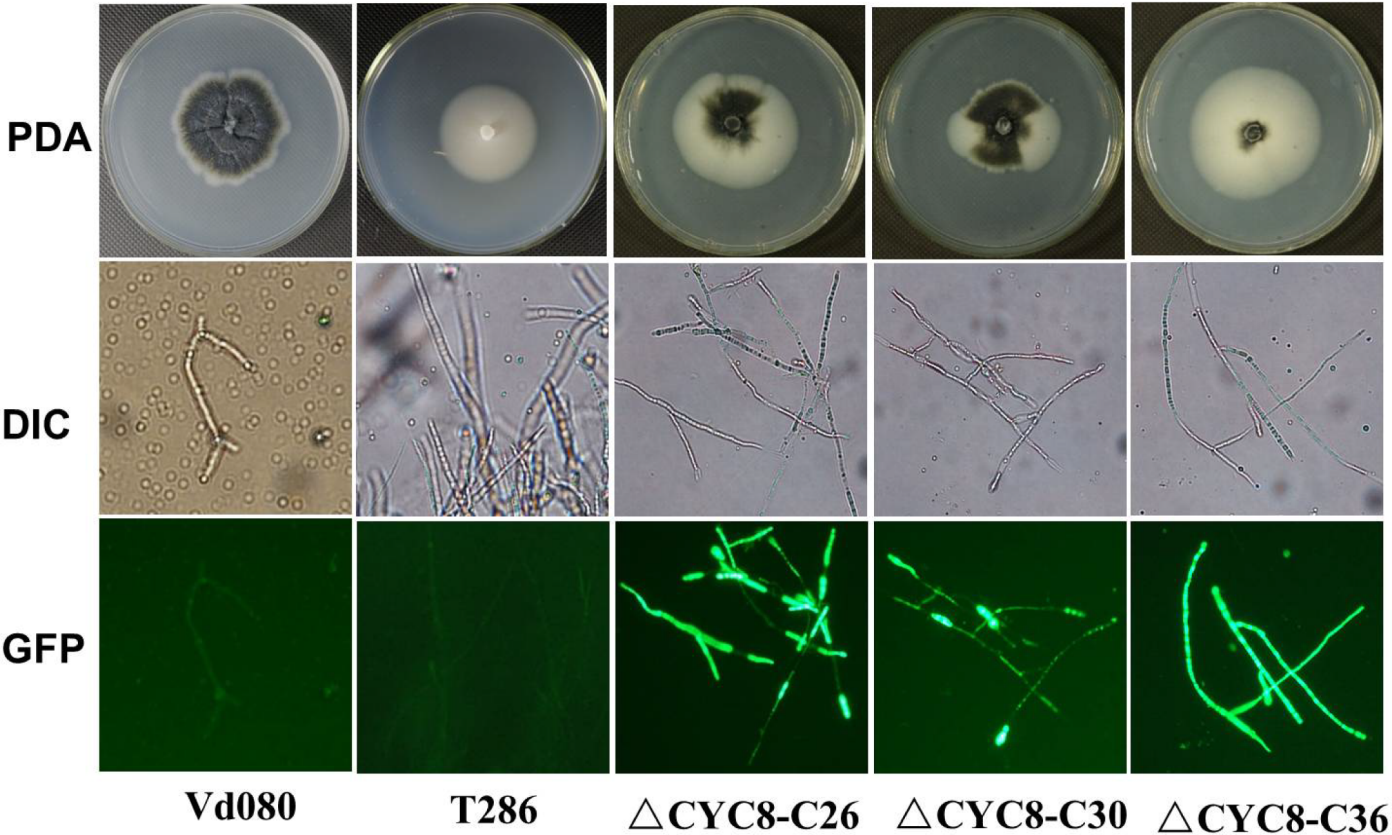
Characteristics of *in vitro* cultures of *Verticillium dahliae* and microscopic observations of complemented mutant strains *ΔCYC8*-C. The comparisons were conducted with the wild-type strain Vd080, the original *VdCYC8* insertional mutant strain T286 and complemented mutants which were tagged with a green fluorescent protein (GFP) gene.

The functional complementation fragment (about 4.3kb in all) was amplified from DNA of wild type strain Vd080 using primers CYC8COM-F/CYC8COM-R and integrated into the binary vector pSULPH-gfp. *A*. *tumefaciens* isolate AGL1 carrying the *CYC8* complementation vector was introduced into the *VdCYC8* disruption mutant strain T286. Fifteen positive complemented mutants were generated, which included both the complementation fragment and chlorimuron cassette (Fig 4A and 4B). Afterwards, three genetic-stability and single-spore-purified mutant strains Δ*CYC8*-C26, Δ*CYC8*-C30 and Δ*CYC8*-C36 were selected for target gene expression analysis and fluorescence observation. *VdCYC8* was transcribed in all of the tested complemented mutant strains. However, relative expression of *VdCYC8* was higher in Δ*CYC8*-C26 than the other two *VdCYC8*-complemented mutants, and showed no significant difference with the expression level observed for the wild type strain Vd080 (Fig 4C). Moreover, the mycelia of all the Δ*CYC8*-C mutants developed normally and exhibited strong green fluorescence under fluorescence microscopy (Fig 5).

Additionally, the result of Southern blot showed that there was no blotting signal of probe *VdCYC8* in Δ*CYC8*-45, Δ*CYC8*-55, Δ*CYC8*-56 and T286, strong signals were present in Δ*CYC8*-C26, Δ*CYC8*-C30 and Δ*CYC8*-C36 and wild type strain Vd080 (S4 Fig).

### *VdCYC8* plays critical roles in melanin accumulation, microsclerotia production, and sporulation

On PDA, deletion mutants of *VdCYC8* exhibited white colonies that were delayed in radial growth, lacked melanin, and produced no microsclerotia (Fig 3D). The average growth rate of *VdCYC8* deleted mutants was 2.56±0.17 mm/d, significantly slower than the wild type Vd080 strain at 4.00±0.10 mm/d (Table 3). For the *VdCYC8*-complemented mutants, fewer microsclerotia appeared than were observed for the wild type strain Vd080, suggesting only partial recovery of the ability of the complemented strain to produce microsclerotia (Fig 5). However, there were no obvious differences in growth rate between the *CYC8* complementary mutants and the wild type strain Vd080 (Table 3).

**Table 3:** Analysis of spore yield and radial growth for Vd080 and its mutants.

Isolate	Spore yield (×10^6^CFU/mL)			Growth rate (mm/d)
	6dpi	8dpi	14dpi	
Vd080	60.50±6.00a*	46.50±11.50a	28.00±21.00a	4.00±0.10a
T286	1.95±0.71b	3.00±0.46b	3.02±1.65b	2.77±0.11b
*ΔCYC8*-45	5.85±1.15c	3.27±0.24b	4.08±1.00bc	2.40±0.04b
*ΔCYC8*-55	5.13±3.25c	4.62±1.28c	2.98±1.31b	2.83±0.15b
*ΔCYC8*-56	1.53±0.46b	4.40±0.23bc	5.70±0.63c	2.46±0.17b
*ΔCYC8*-C26	61.33±4.20a	64.17±12.29a	47.83±9.73d	3.90±0.42a
*ΔCYC8*-C30	41.25±14.19d	43.17±14.50a	36.67±14.50a	4.38±0.16a
*ΔCYC8*-C36	42.08±18.25a	41.17±8.11a	35.83±13.68a	4.50±0.09a

* The subscript letters mean significant difference between mutant and wild-type strain based on the least significant difference test (*P < 0*.*05*).

Similar to the T-DNA insertional transformant T286, *VdCYC8* deletion strains Δ*CYC8*-45, Δ*CYC8*-55, and Δ*CYC8*-56 exhibited sharply reduced sporulation. Following six days of growth, the concentration of wild type Vd080 was 6.0 × 10^7^ conidia/mL, whereas the densities of *VdCYC8* deletion mutant strains Δ*CYC8*-45, Δ*CYC8*-55, and Δ*CYC8*-56 were 5.9 × 10^6^ conidia/mL, 5.1 × 10^6^ CFU /mL and 1.5 × 10^6^ conidia/mL, respectively. Compared with Vd080, the sporulation of the *CYC8* deletion mutants was significantly reduced by one order of magnitude. However, in each of the three *VdCYC8*complemented mutants, Δ*CYC8*-C26, Δ*CYC8*-C30, and Δ*CYC8*-C36, sporulation levels were recovered and the average biomass was in the same order of magnitude as wild type strain Vd080. At 8 14 days of growth, the strains sporulation was consistent among strains, with only slight variation (Table 3). No significant differences in conidia germination were observed between *VdCYC8* deletion mutant strains and Vd080 (data not shown).

### *VdCYC8* is required for virulence on cotton

Pathogenicity assays were conducted to assess the potential contribution of *VdCYC8* to the virulence of *V*. *dahliae*, strain Vd080. Pathogenicity assays were conducted on cotton seedlings following a root-dip inoculation method [39]. The wild type Vd080, Δ*CYC8* and Δ*CYC8* complemented strains led to initial symptoms at various times post-inoculation, and with diverse disease progression. Cotton plants infected with the wild type strain Vd080 exhibited typical leaf necrosis and wilting at 7 dpi, and the disease on these plants progressed rapidly. However, the plants inoculated with deletion mutants Δ*CYC8*-45, Δ*CYC8*-55 and Δ*CYC8*-56 did not show noticeable symptoms until 12 dpi. Likewise, the T-DNA insertional mutant strain, T286, caused first visible wilt disease symptoms at 13 dpi. The *VdCYC8-*complemented strains *ΔCYC8*-C26, *ΔCYC8*-C30 and *ΔCYC8*-C36 caused wilting symptoms at 9 dpi, comparable to the findings observed following inoculation with the wild type strain Vd080.

**Fig 6:**
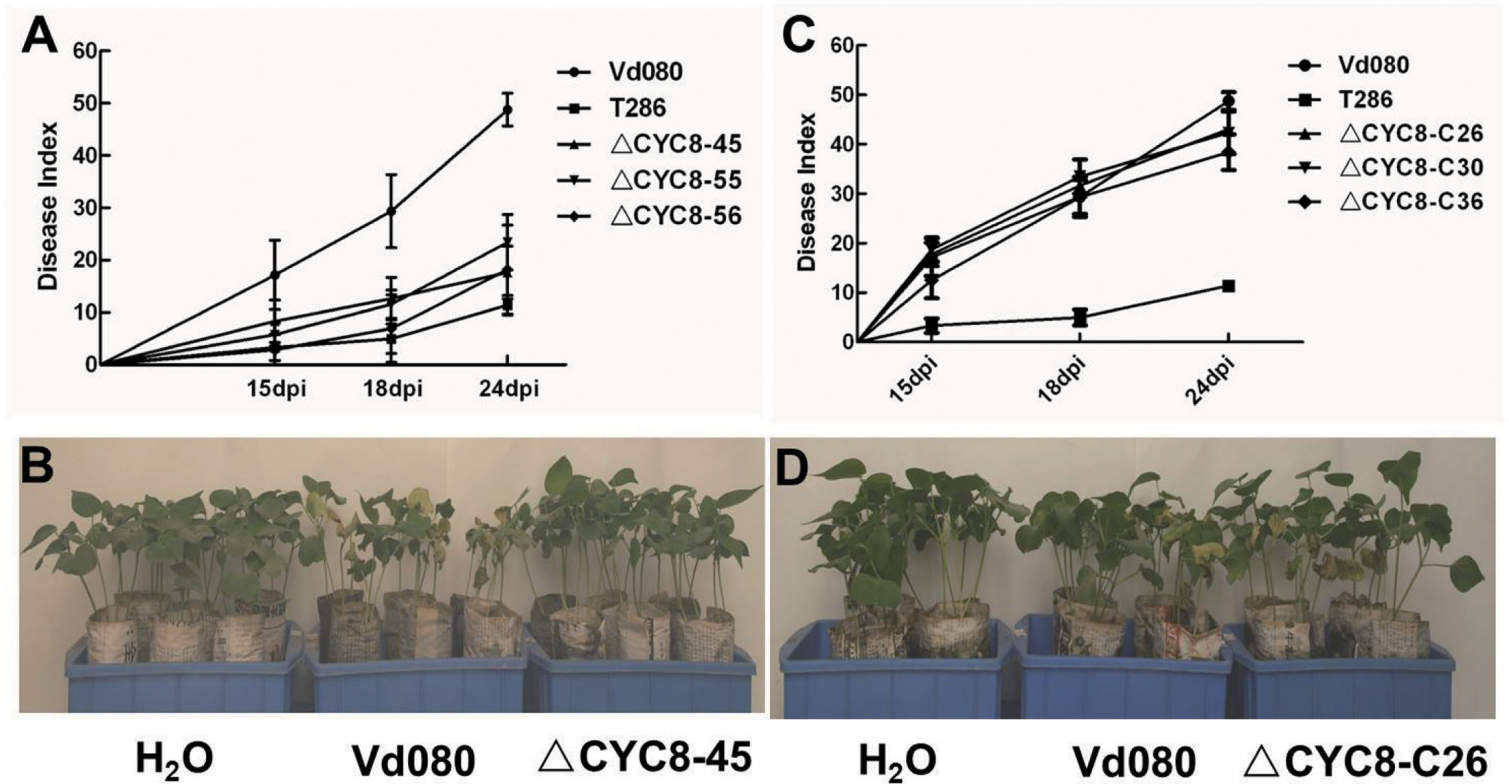
Disease severity analysis of cotton seedlings inoculated with wild type strain Vd080 of *Verticillium dahliae*, and *VdCYC8* mutant strains. **A)** Disease progress curves of cotton plants inoculated with wild-type Vd080, T-DNA insertional mutant T286, and strains Δ*CYC8*-45, Δ*CYC8*-55, and Δ*CYC8*-56. **B)** Disease symptoms of cotton plants inoculated with strains Vd080 and Δ*CYC8*-45. **C)** Disease progress curves of cotton plants inoculated with wild-type Vd080, T-DNA insertional mutant T286, and wild-type complemented strains Δ*CYC8*-C26, Δ*CYC8*-C30 and Δ*CYC8*-C36. **D)** Disease symptoms of cotton plants inoculated with wild type strain Vd080, and the complemented strain Δ*CYC8*-C.

At 24 dpi, 92.2% of the cotton plants inoculated with the wild type Vd080 showed severe symptoms, and a 39.9% mortality rate and a DI value of 48.79±3.11 was recorded. In contrast, for the *VdCYC8* deletion mutant strains Δ*CYC8*-45, Δ*CYC8*-55, and Δ*CYC8*-56 reduced pathogenicity with average DI values of 17.7, 23.4 and 18.2 at 24 dpi, respectively. These values represent reductions of 63.7%, 51.8% and 62.7% in strains Δ*CYC8*-45, Δ*CYC8*-55, and Δ*CYC8*-56, respectively, in contrast to the value recorded for the wild type *V*. *dahliae* strain Vd080 (DI = 48.8). In addition, there was no significant difference in virulence between deletion mutants Δ*CYC8*-45, Δ*CYC8*-55, and Δ*CYC8*-56 and the original *CYC8* insertional mutant strain T286 (Fig 6A and 6B). The reintroduction of *VdCYC8* to Δ*CYC8* strains restored near wild-type levels of virulence in each of the complemented mutant strains Δ*CYC8*-C26, Δ*CYC8*-C30 and Δ*CYC8*-C36. At 18 dpi, plants infected with *VdCYC8-*complemented mutant strains showed statistically similar disease levels to those observed when the plants were inoculated with the wild type strain Vd080. At 24 dpi, the *VdCYC8*-complemented strain Δ*CYC8*-C26 exhibited strong virulence. Although the strains Δ*CYC8*-C30 and Δ*CYC8*-C36 were less virulent than Vd080, the strains Δ*CYC8*-C30 and Δ*CYC8*-C36 showed significantly higher DI values than the *VdCYC8* deletion mutant strains (Fig 6C and 6D). Interestingly, virulence observed following inoculation of complemented mutant strains was correlated with the *VdCYC8* gene expression level (Fig 4C). That is, *VdCYC8* gene expression was highest in strain *ΔCYC8*-C26, relative to *VdCYC8*, and strain *ΔCYC8*-C26 showed strongest virulence to cotton seedlings (Fig 6C).

### *VdCYC8* regulates transcription of several genes involved in melanin production and microsclerotia formation in *V*. *dahliae*

**Fig 7:**
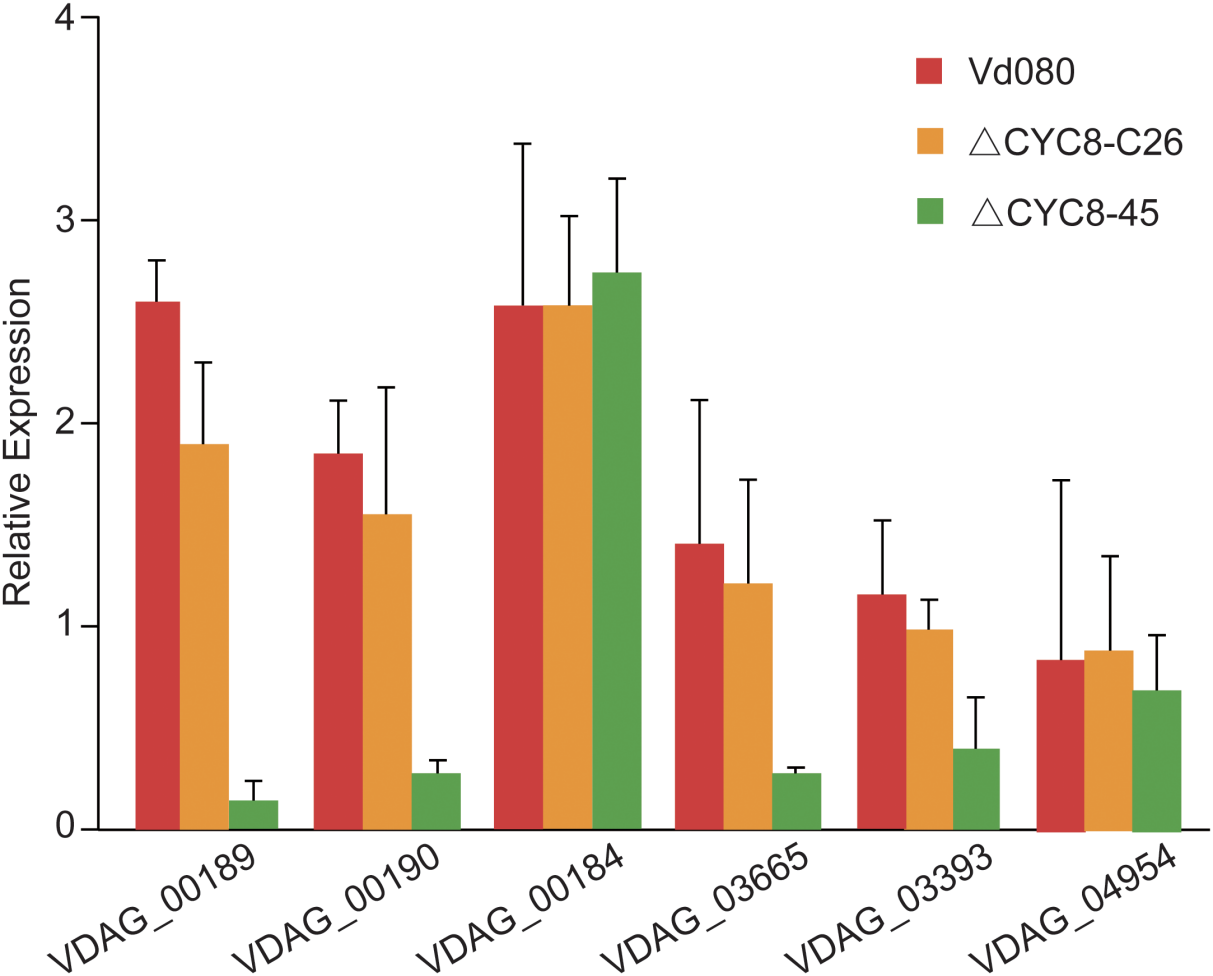
Transcriptional expression analysis of genes expressed during microsclerotia formation in *Verticillium dahliae* in the wild-type isolate Vd080, the deletion mutant Δ*CYC8*-45, and wild type-complemented mutant strain Δ*CYC8*-C26. The genes highly expressed during microsclerotia formation were previously identified [40], and the primer sets for their amplification in this study are listed in Table 2. The *V*. *dahliae β-tubulin* (Bt), amplified using primers VerBt-F/R (see Materials and Methods), was used in relative expression analyses.

As mentioned earlier, the Δ*VdCYC8* strains of *V*. *dahliae* strain Vd080 produced no melanin or microsclerotia, but this phenotype could be partially restored in each of the *VdCYC8*—complemented mutants. To further investigate the role of *VdCYC8* in melanin biosynthesis or microsclerotia biogenesis, the transcriptional expression of *VDAG_00189* (encoding laccase), *VDAG_00190* (conidial yellow pigment biosynthesis polyketide synthase), *VDAG_00184* (amino acid adenylation/polyketide synthase), *VDAG_03665* (tetrahydroxy-naphthalene reductase), *VDAG_03393* (scytalone dehydratase), and *VDAG_04954* (pigment biosynthesis protein) was evaluated in the wild type strain Vd080, the complemented CYC8 mutant strain Δ*CYC8*-C26, and the knockout mutant ΔCYC8-45. Compared with the expression levels observed in the wild type Vd080 and Δ*CYC8*-C26 strains, the reduced expression of *VDAG_00189*, *VDAG_00190*, *VDAG_03665*, and *VDAG_03393* were each reduced in the absence of Δ*VdCYC8*, and showed drastic decline in ΔCYC8-45 (Fig 7). However, deletion mutation of CYC8 had no effect on the expression of *VDAG_00184* and *VDAG_04954* as there were no observed transcriptional alterations for *VDAG_00184* and *VDAG_04954* among the tested strains (Fig 7).

## Discussion

In this study, we examined the function of *VdCYC8* of *Verticillium dahliae*, encoding a homolog of the fungal glucose repression mediator protein CYC8. The homologs of *VdCYC8* can be transcriptional repressors, regulating many developmental and metabolic processes in yeast [26, 27]. The results of this study indicate that VdCYC8 is involved in microsclerotia formation, conidia production, speed of growth in culture, and virulence on cotton. *V*. *dahliae* deletion mutants of *CYC8* (Δ*CYC8*) lost the ability to produce melanin and microsclerotia, and also exhibited decreased sporulation, stunted growth, and sharply reduced pathogenicity on cotton seedlings. Compared with the wild type strain Vd080, the Δ*CYC8* strains showed a 5-day delay in the development of typical Verticillium wilt symptoms. These phenotypic differences in the Δ*CYC8* strain were partially or completely restored in complementary mutant strains Δ*CYC8-C*.

Mirosclerotia are long-term survival structures, and are the primary inoculum for originating Verticillium wilt disease. They are considered to be an important model for exploring the initial penetration and pathogenicity mechanisms in *V*. *dahliae* [2, 3, 42, 43]. Entire genome-wide expression profiles were conducted to screen differentially expressed genes by comparing cDNA libraries between germinated and non-germinated microsclerotia. One hundred and four genes were expressed exclusively in the germinated microsclerotia, including those encoding the G-protein coupled receptor, cyclopentanone, glucosidase, alcohol dehydrogenase, and others [44]. Conidia serve to transport and disperse the pathogen in the host xylem vessels [45], procuring nutrients from the host for successful colonization and fungal fitness [46]. Enhancement of desiccation tolerance could prolong survival and accelerate their proliferation within the host [47]. In summary, microsclerotia and conidia are critical propagules for infection and colonization of host plants and are key components of *V*. *dahliae* pathogenesis.

In the current study, the *VdCYC8* complemented mutant strains exhibited partial restoration of the wild type phenotype. We speculated that the transcriptional expression level was the primary factor affecting the complementation efficiency, and RT-qPCR was conducted to evaluate the *VdCYC8* expression level in each complementary mutant. Several protocols are available for gene complementation in *V*. *dahliae* including overexpression of target genes [19, 48, 49], functional copy reintroduction [13, 16, 17] and exogenous protein expression [47]. Complementary location is random in the deleted mutants without homologous recombination. Most mutants were ectopic insertion instead of specific locus complementation. RT-qPCR was practical in this study to assess the expression level of target genes, and provided data for expression vs phenotype correlation analyses.

The findings herein confirm important roles of *VdCYC8* in virulence and development, similar to roles ascribed to the *VdCYC8* homolog in morphogenesis and virulence in *C*. *albicans* [39]. The defect in *VdCYC8* clearly compromised wild type levels of microsclerotial formation, and we speculate that defect directly or indirectly affects virulence in *V*. *dahliae*. As reported in other studies, there is an important association between microsclerotia formation and developmental processes that are required for virulence in *V*. *dahliae* [19–21].

Recent analyses microsclerotia biogenesis and melanin synthesis in *V*. *dahliae* by RNA-seq or microarray analysis has revealed numerous target genes that are differentially expressed in microsclerotial and amicrosclerotial cultures [40]. Six candidate genes (*VDAG_00189*, *VDAG_00190*, *VDAG_00184*, *VDAG_03665*, *VDAG_03393*, *VDAG_04954*) involved in melanin synthesis or microsclerotia formation were selected as indicators to investigate the pathways related to *VdCYC8*. With the absence of *VdCYC8*, the transcriptional expression of *VDAG_00189* (encoding a laccase), *VDAG_00190* (conidial yellow pigment biosynthesis polyketide synthase), *VDAG_03665* (tetrahydroxy- naphthalene reductase) and *VDAG_03393* (scytalone dehydratase) showed significant reductions (Fig 7). This indicated that *VdCYC8* was tightly linked with the pathways mentioned above. Interestingly, unlike *VDAG_00190*, *VDAG_00184* (amino acid adenylation/polyketide synthase), which also shared homology with genes from *Penicillium marneffei* that were involved in melanin biosynthesis [50], was not regulated by *VdCYC8* in *V*. *dahliae*. Another gene, *VDAG_04954*, previously identified as up-regulated by RNA sequencing and microarray analysis during microsclerotia development [15, 40], was independent of *VdCYC8* regulation in this study.

The results presented herein demonstrate the complexity of melanin production and microsclerotia formation in fungi [51, 52]. *VdCYC8* was involved in several pathways during microsclerotia formation. Further studies are underway to determine the signal transduction pathway of this pivotal, multi-faceted gene in *V*. *dahliae*. The roles of *VdCYC8* in host penetration, colonization of vascular tissues, and infection will be addressed in future studies, and this knowledge may lead to additional insights on the disruption of signaling for the control of this pathogen.

## Supporting Information

S1 Fig Schematic diagrams of pGKO_2_-*CYC8* knock out vector construction for replacement of *VdCYC8* in *Verticillium dahliae*, strain Vd80, with a hygromycin-resistance gene cassette. **A)** Acquisition of the *CYC8* fusion fragment containing the hygromycin-resistance gene cassette (Hyg cassette) using the *CYC8*-Hyg cassette overlap primers P3 and P4 in combination with *CYC8*-flanking primers P1 and P6, followed by fusion PCR. **B)** Flowchart of pGKO_2_-CYC8 construction, mediated by a gateway reaction involving homologous recombination at attP sites, located between the T-DNA left border (LB) and right border (RB).
(JPG)

S2 Fig Phylogenetic analyses of protein VdCYC8 of *Verticillium dahliae* in relation with homologs from other fungal species. The amino acid sequences of CYC8 from 29 fungi were aligned using Clustal_X and the phylogenetic tree was constructed using the Mega v.5.1 software with the neighbor-joining method. Bootstrap percentages over 50% are indicated at the nodes.
(PNG)

S3 Fig Electrophoresis of *CYC8* DNA fragment production and fusion PCR for vector construction. Lanes 1–5 indicate the DNA fragment flanking the 5’ of *VdCYC8* (UP), the DNA fragment flanking the 5’ of *VdCYC8* (DOWN), hygromycin resistance cassette (HPH), PCR fusion product, and nested PCR product, respectively. The molecular weight marker (M) is 1kb ladder.
(JPG)

S4 Fig Positive mutants verification with Southern blot. The molecular weight marker (M) is 1kb ladder. Blank (lane 1), Δ*CYC8*-45 (lane 2), Δ*CYC8*-55 (lane 3), Δ*CYC8*-56 (lane 4), Δ*CYC8*-C26 (lane 5), Δ*CYC8*-C30 (lane 6), Δ*CYC8*-C36 (lane 7), T286 (lane 8), Vd080 (lane 9), respectively.
(JPG)
