# Supplementary figures and images for: VdCYC8, Encoding CYC8 Glucose Repression Mediator Protein, Is Required for Microsclerotia Formation and Full Virulence in Verticillium dahliae
Source: PLoS One. 2015 Dec 3;10(12):e0144020. doi: 10.1371/journal.pone.0144020 (PMC4669128; doi:10.1371/journal.pone.0144020)

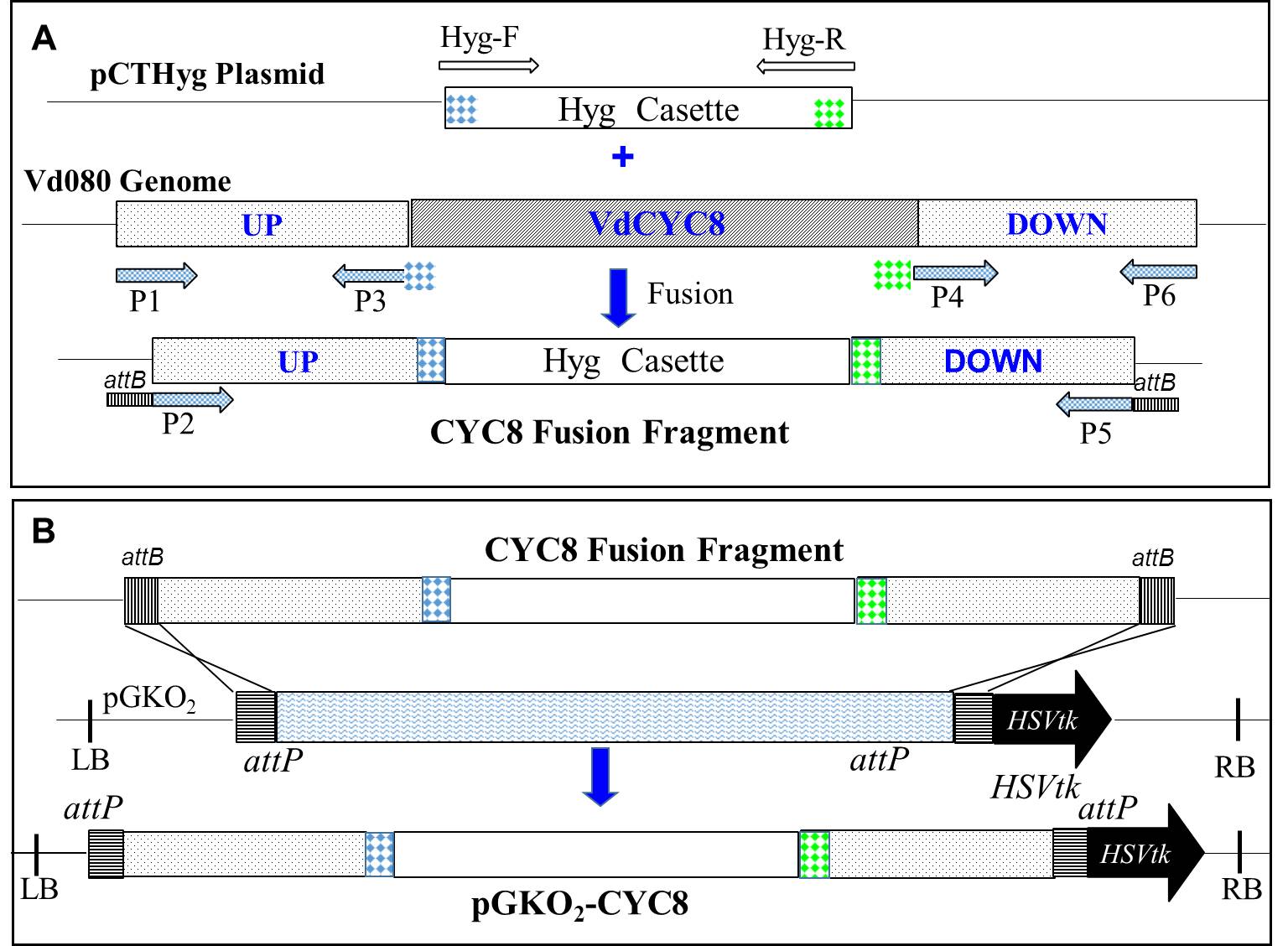

Supplement: S1 Fig — A) Acquisition of the CYC8 fusion fragment containing the hygromycin-resistance gene cassette (Hyg cassette) using the CYC8-Hyg cassette overlap primers P3 and P4 in combination with CYC8-flanking primers P1 and P6, followed by fusion PCR. B) Flowchart of pGKO2-CYC8 construction, mediated by a gateway reaction involving homologous recombination at attP sites, located between the T-DNA left border (LB) and right border (RB). (JPG) [file pone.0144020.s001.jpg]

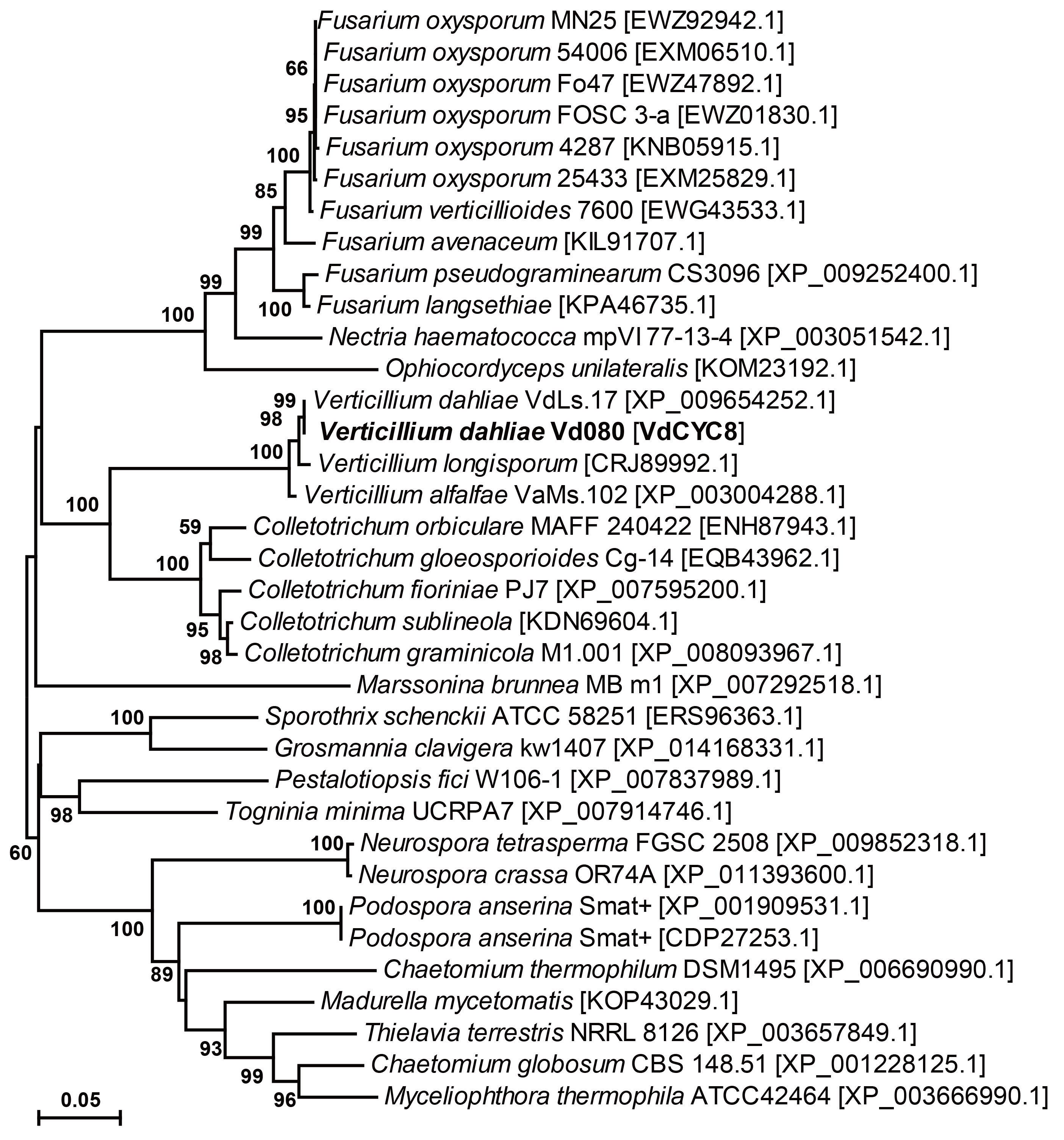

Supplement: S2 Fig — The amino acid sequences of CYC8 from 29 fungi were aligned using Clustal_X and the phylogenetic tree was constructed using the Mega v.5.1 software with the neighbor-joining method. Bootstrap percentages over 50% are indicated at the nodes. (PNG) [file pone.0144020.s002.png]

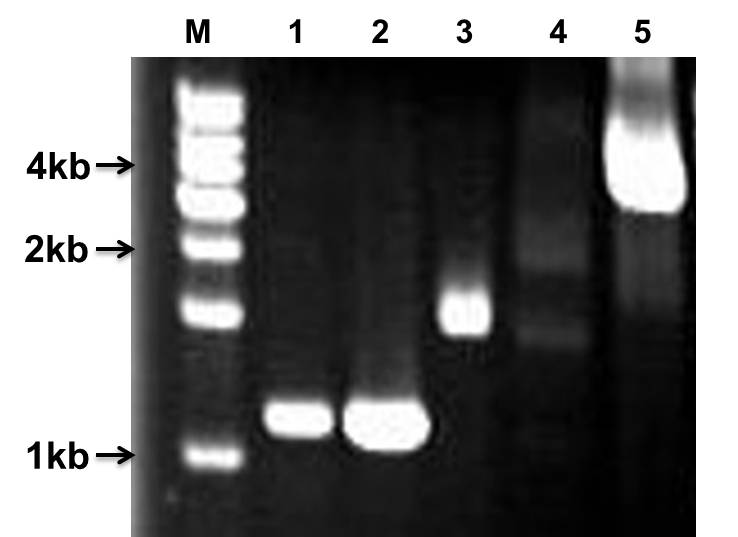

Supplement: S3 Fig — Lanes 1–5 indicate the DNA fragment flanking the 5’ of VdCYC8 (UP), the DNA fragment flanking the 5’ of VdCYC8 (DOWN), hygromycin resistance cassette (HPH), PCR fusion product, and nested PCR product, respectively. The molecular weight marker (M) is 1kb ladder. (JPG) [file pone.0144020.s003.jpg]

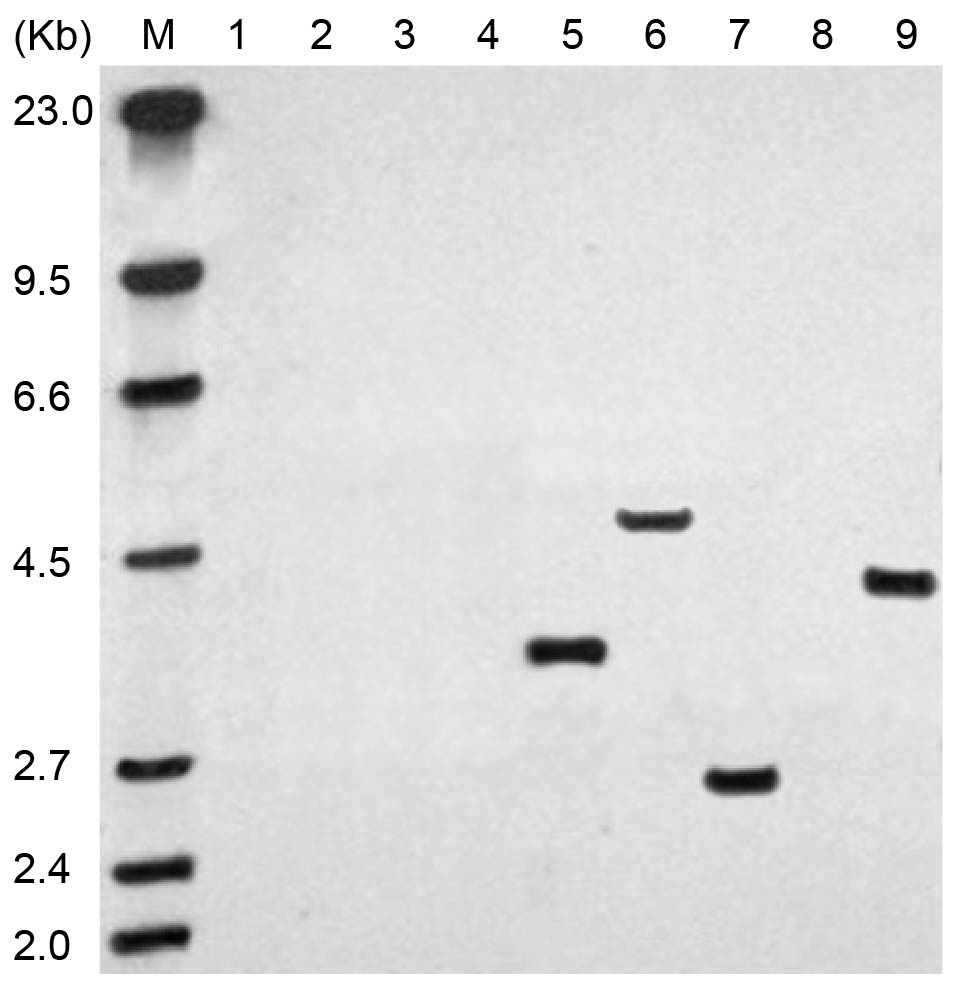

Supplement: S4 Fig — The molecular weight marker (M) is 1kb ladder. Blank (lane 1), ΔCYC8-45 (lane 2), ΔCYC8-55 (lane 3), ΔCYC8-56 (lane 4), ΔCYC8-C26 (lane 5), ΔCYC8-C30 (lane 6), ΔCYC8-C36 (lane 7), T286 (lane 8), Vd080 (lane 9), respectively. (JPG) [file pone.0144020.s004.jpg]
